# Experimental Investigation on the Formability of Al-Mg Alloy 5052 Sheet by Tensile and Cupping Test

**DOI:** 10.3390/ma15248949

**Published:** 2022-12-14

**Authors:** Hongmei He, Tao Yang, Yi Ren, Yi Peng, Song Xue, Lixuan Zheng

**Affiliations:** 1 School of Manufacturing of Science and Engineering, Southwest University of Science and Technology, Mianyang 621010, China; 2 Key Laboratory of Testing Technology for Manufacturing Process, Ministry of Education, Mianyang 621010, China

**Keywords:** Al-Mg alloy, sheet forming, formability, cupping, tensile testing

## Abstract

Aiming at the enhancement of the lightweight formability potential of aluminum alloy, the bulging and tensile properties of a 5052 Aluminum alloy sheet were tested on a microcomputer controlled sheet metal forming tester and tensile testing machine. The effects of different blank holder force, punch velocity and lubrication conditions were investigated on bulging properties by the experimental analysis. The cupping values (Erichsen Cupping Index: IE) of sheets with a thickness of 1.2 mm at room temperature were obtained under different process parameters. Meanwhile, the anisotropic property of the material was analyzed in different rolling directions. The results show that the sheet cupping values increase with the increase of blank holder force and punch velocity, and the stress state was changed due to the changing of the blank holder force and strain rate. Moreover, the use of lubricating conditions with a lower coefficient of friction allows the sheet to exhibit a larger cupping value. The effect of rolling direction on the anisotropy of 5052 aluminum alloy sheet is distinct, which means in the aluminum alloy sheet forming process the anisotropy factor should be carefully considered.

## 1. Introduction

With the development of environment pollution and the decrease of petrol sources all over the world, economic and social development are greatly affected. Useful and sustainable methods were proposed by some researchers to reduce the environmental pollution and improve the efficiency of petrol. For example, reducing the weight of vehicles used in aviation and aerospace [1,2]. In order to achieve this goal, some light metal materials were used to produce some parts in the structure and body of the vehicles for aviation and aerospace, such as aluminum alloy and magnesium alloy materials [3,4,5]. As we all know, aluminum alloy sheets are one of the cheaper light metal materials, and they are widely used in vehicles for the aviation and aerospace industries to reduce their weight. Uses include the oil pans of engines, the outer housings of motors, the fuel tanks of rockets and the skin panels of airplanes [6,7,8].

Aluminum (Al) is the lightest metal that can be employed in structural applications when it is alloyed with other elements. Aluminum alloy material is a new type of alloy material that emerged in the 1980s, which is widely used due to its lower density, high strength-to-weight ratio and the good corrosion resistance. At present, aluminum alloy material has been developed into several different series, among them, the 5052 aluminum alloy is one of the aluminum–magnesium alloys. Magnesium is the strengthening element, and it could improve the properties of aluminum alloy material. The 5052 aluminum alloy accounts for a large proportion of the vehicle industry, and it plays an important role in the process of making vehicles lightweight [9,10,11]. However, aluminum alloy sheets have a lower formability compared with the conventional automotive sheet alloys at room temperature. For example, the elongation of aluminum alloy sheet material is only a half of the conventional steel and the formability of deep-drawing is only two-thirds of conventional steel [1,12,13]. Therefore, it is of great significance to study the formability of aluminum alloy sheets.

Stamping is a common processing and forming method of metal sheets. It utilizes the plastic deformation ability of metal materials to make metal sheets that are formed or separated from the materials using the action of an external load. Finally, the shape and size of products is determined. The plastic formability of metal sheets is seriously affected by the different forming conditions, so the rationality of stamping schemes was the key to improve the forming limits of aluminum alloy sheets, which can reduce the time of the casting process [14,15]. In general, the plastic property of the material was tested by tensile testing and cupping testing, and the forming properties could be analyzed by this test value. The Erichsen Cupping Test is an effective method which can evaluate the plastic and forming limit of the material [16].

Singh et al. [17] studied the formability of AA1200 aluminum sheets by the Erichsen Cupping test method, and the study results showed that the formability of aluminum alloy sheets could be increased by controlling the annealing temperature in the process of heat treatment and changing the chemical composition of material. Dewang et al. [9] studied the effect of the stretch flanging process by different initial flange length, punch-die clearance and blank holder force. Pranavi et al. [18] studied the forming limits of AA6061 aluminum alloy on the different punching radius, lubricating conditions, die radius and blank holder forces on deep drawing process conditions. Aminzahed et al. [19,20,21,22] studied the effect of blank holder force on forming properties in the micro-drawing process of aluminum alloy sheets. The results showed that the punch force and spring-back of material could be affected seriously by controlling the blank-holder force in the stamping process. Additionally, the punch force and spring-back of material was reduced by improving the blank holder force. Meanwhile, their study showed that the spring-back of material was reduced by increased the thickness of material in the drawing process. The above investigations and analysis provide some fundamentals to study the forming limits of aluminum alloy sheet material, and can provide some guidance for this article to study the formability of a 5052 aluminum alloy sheet.

However, due to the control accuracy of the testing equipment, previous research was more about the trend of material forming performance. A more comprehensive study on the formability of a 5052 aluminum alloy sheet will help to better understand the forming characteristics of the materials and provide favorable conditions for the designing and processing.

In this paper, the 5052 aluminum alloy sheet was the study material, which was studied by tensile testing and cupping testing, and many forming conditions were concerned in the experimental process. The research work was carried out from the mechanical behavior of the material deformation aspect and the effect of direct forming aspect. The study results will help other researchers understand the material properties better and improve the application of materials.

## 2. Material and Experimental Methods

### 2.1. Material

In this study, the test material selected was the 5052-H24 Al–Mg alloy sheet, which was the commercial aluminum alloy material. According to the requirement standard of GB/T4156-2020 [23] (equivalent to ISO 20482-2013), the test specimens were cut into 100 mm × 100 mm and the thickness of material is 1.2 mm. The chemical composition and mechanical properties of the aluminum alloy sheet is listed in Table 1 and Table 2.

### 2.2. Experimental Methods 

#### 2.2.1. Tensile Testing

Tensile testing is the basic engineering experiment to test the formability of metal material and the properties of metal can be obtained by using this method in the testing process. Properties such as the yield strength, strain at break, ultimate tensile strength and Young’s modulus can be determined. The forming limit of the material was obtained by the properties of the metal [25,26].

#### 2.2.2. Cupping Test

**Figure 1:**
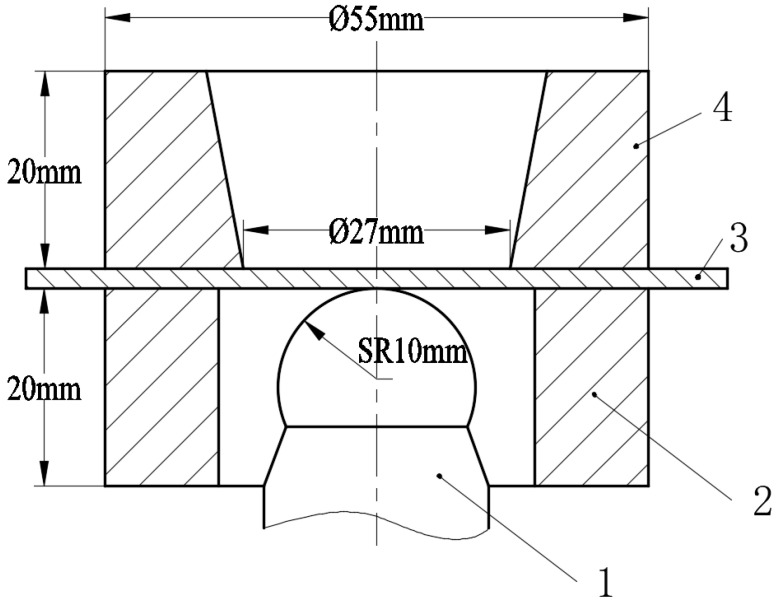
The schematic diagram of cupping test device. 1. Punch; 2. Lower holder; 3. Aluminum sheet; 4. Upper holder.

**Figure 2:**
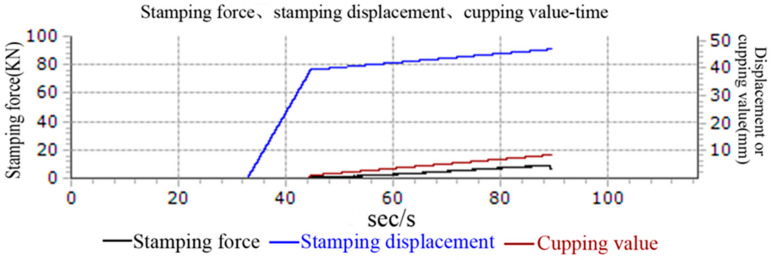
The cupping test curves.

The metal cupping test is usually used to analyze the bulging limit of a metal sheet [27]. The schematic diagram of cupping test device was showed in Figure 1. The bulging limit of metal sheet was depended on the local plastic flowing ability in the bulging area. However, the local plastic flowing ability of material was affected by the blank-holder force, friction coefficients and punch velocity. The cupping test value was called the IE (Erichsen Cupping Index) value of the testing material, which was determined by the distance from punch touch to the sheet until it is broken. The larger the IE value, the better the bulging performance of the material [28]. The input signal is analyzed and processed by the computer, and the stamping force, stamping displacement and cupping value with time curves are shown in Figure 2. The cupping test process: During the clamping process, the punch does not move upwards and the stamping displacement is 0. When the clamping force reaches the specified value, the punch begins to move upward, meanwhile, the stamping displacement value appears. Stamping force and cupping values begin to appear when the punch touches the sheet, so the figure shows that two curves appear and rise at the same starting point. The stamping force drops rapidly when the sheet reaches a state of instability. 

In this test, the test was carried out on a microcomputer controlled sheet forming test machine. (The maximum allowable stamping load of the equipment is 300 kN, the maximum clamping load is 300 kN and the equipment accuracy is 0.02 mm). The test termination criteria are set to when the stamping force drops to 90% of the maximum stamping force.

## 3. Results and Discussion

### 3.1. Tensile Testing in Different Directions

**Figure 3:**
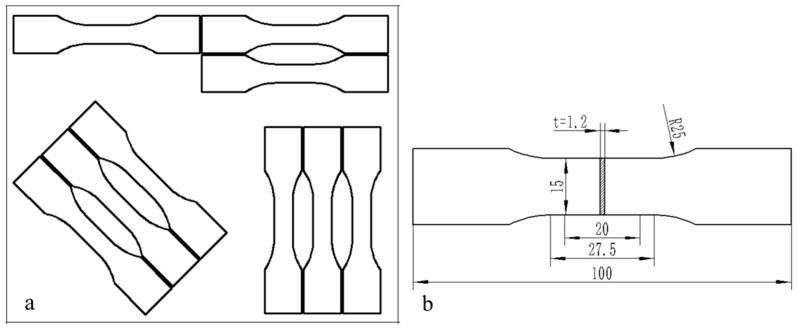
The tensile testing samples. (**a**) Sampling scheme. (**b**) Tensile specimen.

**Figure 4:**
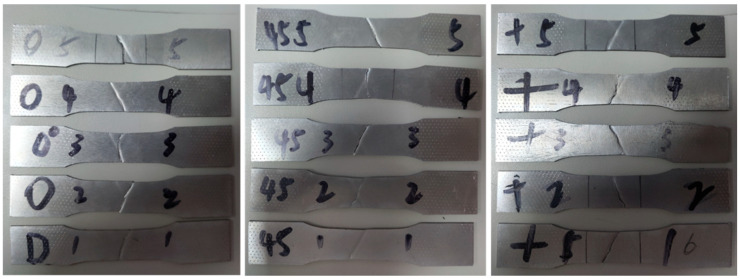
The samples of tensile testing in different directions.

According to the GB/T 228-2002 metallic materials-test pieces for tensile testing [29], the specimens were cut from three directions of material (the 45°, 90° and 0° to the side of sheet) as shown in Figure 3a. The size of tensile specimens were prepared as shown in Figure 3b. And the specimens were carried out on a microcomputer controlled sheet tensile testing machine. Each sample was tested five times repeatedly as shown in Figure 4, and then the average value of the five test results was calculated.

The specimens were tested by tensile testing at room temperature and the test values of the yield strength, the percentage elongation and the ultimate tensile strength are shown in Table 3. The results showed that the anisotropy was indicated in different rolling directions of the 5052 aluminum alloy sheet. The minimum yield strength value was obtained at 45°, and it increased substantially in the other two directions. The ultimate tensile strength value was at minimum at 45°, and the percentage elongation value was at minimum at 0°. This was caused by the different mechanical properties that emerged in different rolling directions.

There was great affects from the rolling direction of the aluminum alloy sheet on mechanical properties. The orientations of the grain of the metal material were affected due to the different rolling directions of the aluminum alloy sheet. The different stress was reflected in the forming process of material due to the different directions of grain. Thus, the different formability was obtained in different drawing and rolling directions. Therefore, it is pretty significant that the forming ability of an aluminum alloy sheet was controlled by selecting different drawing directions with the rolling direction of an aluminum alloy sheet.

### 3.2. Cupping Test at Different Blank Holder Forces

**Figure 5:**
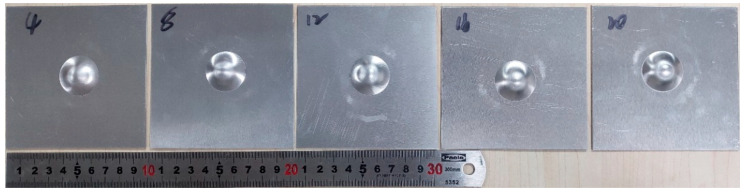
The specimens tested under different blank holder forces.

**Figure 6:**
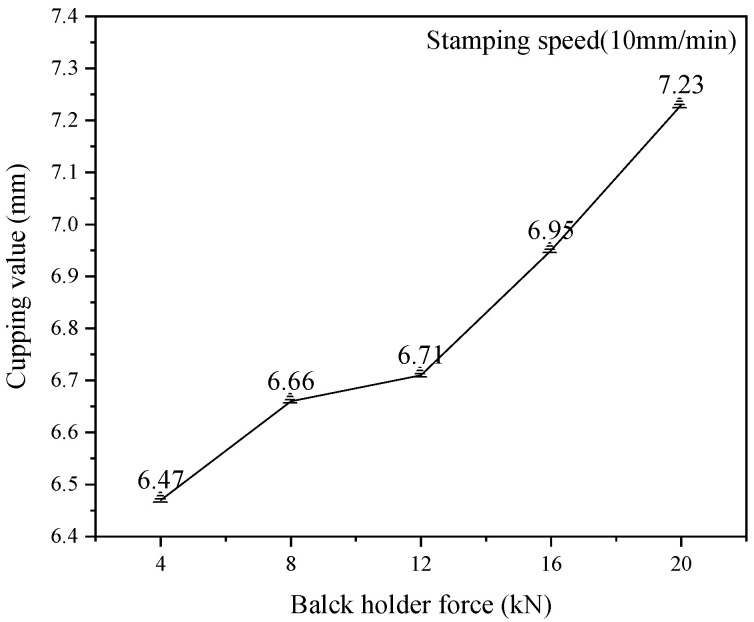
The relationship between blank holder forces and IE values.

In order to analyze the effect of the blank-holder force, the blank holder testing was set five different values (4kN, 8kN, 12kN, 16kN and 20kN) as shown in Figure 5. The testing was carried out on the no lubrication condition, and the stamping speed of the punch was set at 10 mm·min-1. Five specimens made a team, which was tested five times repeatedly using the same method, and the average value was calculated. Finally, the cupping values of the aluminum alloy sheet under the different blank holder loads were obtained, as shown in Figure 6. The results showed that the cupping value increased with the increase of the blank-holder force. In the cupping process, the stress state of aluminum alloy sheet was changed due to the changing of the blank-holder force. The tensile stress was formed in the process when the blank-holder force was increased at 20kN. Thus, the force of the punch was counteracted by the tensile stress of the aluminum alloy sheet in the previous phases of cupping, and the direction of tensile stress was changed with the raised of the punch with the increase of the blank holder force. Therefore, the plastic deformation properties of the sheet can be better improved by selecting suitable blank holder force.

### 3.3. Cupping Test at Different Punch Velocities

**Figure 7:**
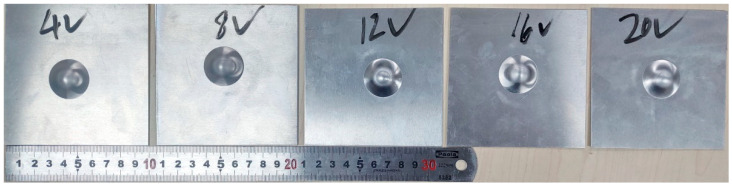
The specimens tested under different punch velocity.

**Figure 8:**
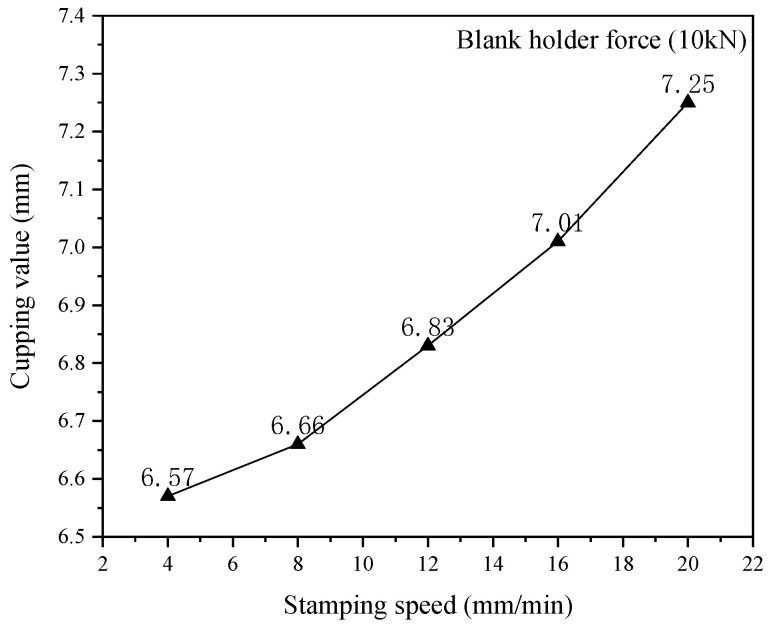
The relationship between punch velocity and IE values.

Strain rate is one of the most influential factors on changing the forming properties of the aluminum alloy sheet. The formability of material in the stamping process has effects on the different strain. The strain rate was replaced by velocity in the test process. Therefore, it was a method to analyze the formability of an aluminum alloy sheet by changing the velocity of punch, and the velocity of punch was set 4, 8, 12, 16 and 20 mm·min^−1^, as shown in Figure 7. The blank holder force of an aluminum alloy sheet was set to 10kN and the lubrication condition was set no lubrication. In this test condition, five specimens made a team, which was tested five times repeatedly using the same method, and the average value was calculated. Finally, the cupping values of the aluminum alloy sheet to the different punch velocities were obtained, as shown in Figure 8.

As can be seen from Figure 8, in the range of the punch velocity, the cupping value was increased with the speed velocity. Plastic deformation was constantly transferred from the strong zone to the weak zone due to the strain-hardening phenomenon in the process of stamping deformation. Under the lower punch velocity, the aluminum alloy continuously flowed from the weak deformation zone to the strong deformation zone. With the increase of punch velocity, a large number of weak deformation zones transferred into strong deformation zones, the hardening state and strength of the deformation zones was increased and the coordination ability of the deformation zone was also improved. Therefore, the punch needs to move to a greater stamping displacement to satisfy the criteria of sheet rupture, that is the cupping values become larger.

### 3.4. Cupping Test under Different Lubrication Conditions

**Figure 9:**
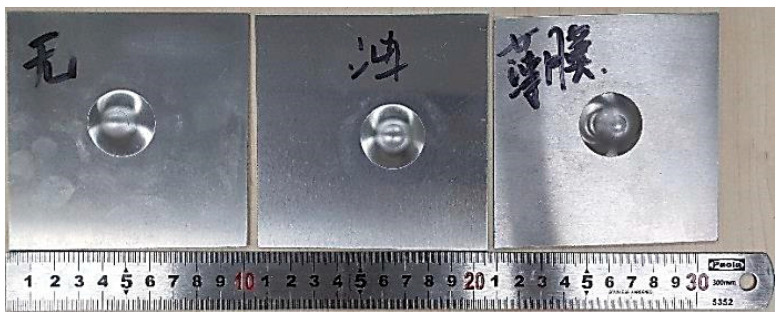
The specimens tested under different lubrication conditions (无 No Lubrication, 油 Oil, 薄膜 Thin-Film).

**Figure 10:**
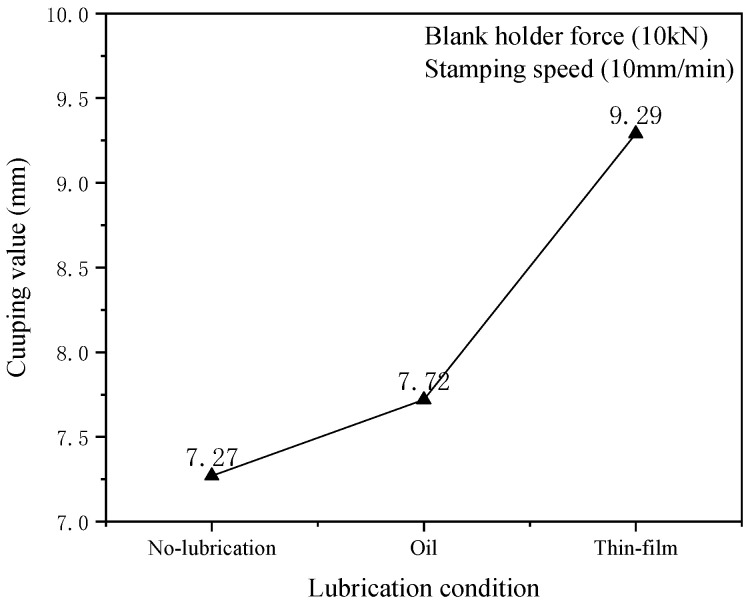
The relationship between lubrication conditions and IE values.

The friction coefficient between punch and plate was the important impact factor. Therefore, it is pretty important to choose different friction coefficients in the experimental process. The lubricating oil was evenly coated on the side where the plate contacts the ball head, or a thin film was pasted on the metal sheet. Then, the punch velocity was set to 10 mm·min^−1^ and the blank holder force was set to 10kN. The 0.8 friction coefficients of Thin-film lubrication were selected and the 0.1 friction coefficients of oil lubrication were selected at room temperature. The cupping experiment was carried out on this test condition and the test results are shown in Figure 9. Five specimens were repeatedly tested under each of the corresponding lubrication conditions, and then the cupping values on the different lubrication conditions are obtained by means of calculating the average value, as shown in Figure 10. In addition, the friction coefficients on different lubrication conditions are given in Table 4.

As can be seen from Figure 10, the flow situation of the material was increased in the forming process due to the changed lubrication conditions, and the bulging properties of the material can be improved by using lubricant. The cupping value of thin-film lubrication is larger than that of oil lubrication. The minimum friction coefficient was obtained and the bulging properties of the 5052 aluminum alloy were the best by using the thin-film lubrication. The flowing ability of the aluminum alloy sheet was improved by reducing the friction coefficient and the uniformity of forming of material was increased due to the better flowing ability. Therefore, suitable lubrication conditions can be used to increase the formability of the 5052 aluminum sheets in practice.

## 4. Conclusions

Friction coefficients, punch velocity and blank-holder force in the sheet-forming process were considered to investigate the bulging properties of a 5052 aluminum alloy sheet using the Erichsen cupping test. The anisotropy of the 5052 aluminum alloy sheet of different rolling directions was analyzed. The achievements of the current study are summarized as follows:

(1) In this study, the maximum blank holder force is 20kN in the experimental process. The biggest cupping value was obtained with this condition. The stress state and the direction of the tensile stress of the aluminum alloy sheet was changed in the cupping process with the increase of blank holder force. The part of force of the punch was counteracted to the tensile stress when the blank holder force reached a higher value. Thus, the formability of an aluminum alloy sheet was improved by increasing the blank-holder force.
(2) Within the specified strain rate, the coordination ability of the deformation and the transformation ability from strong zone to weak zone of material were improved with the increase of the strain rate. Meanwhile, the hardening had effects on the formability of the aluminum alloy sheet in the cupping process. Thus, the bulging property could be improved by increasing the strain rate.
(3) The cupping ability could be improved by reducing the friction coefficients between the punch and aluminum alloy sheet. Therefore, the bulging property of 5052 aluminum sheets could be improved by choosing good lubrication conditions.
(4) The effect of the rolling direction on the anisotropy of a 5052 aluminum alloy sheet is obvious, which indicated that the anisotropy factor should be carefully considered in the aluminum alloy sheet forming process.

## Figures and Tables

**Table 1:** Chemical composition of 5052 Al–Mg alloy (%, mass fraction) [24].

Element	Si	Cu	Mg	Zn	Mn	Cr	Fe	Al
Standard value	≤0.25	≤0.10	2.2~2.8	≤0.1	≤0.10	0.1~0.35	≤0.40	Bal.

**Table 2:** Mechanical properties of 5052 Al–Mg alloy [24].

Item	Tensile Strength (MPa)	Yield Strength (MPa)	Elongation after Fracture (%)
Standard value	210–260	≥130	≥6

**Table 3:** Mechanical properties of sheet metal in each direction.

The Sampled Direction	Yield Strength (MPa)	Ultimate Tensile Strength (MPa)	Percentage Elongation (%)
0°	88	132	14.0
45°	50	80	17.5
90°	83	118	20

**Table 4:** Friction coefficients of sheet under different lubrication conditions [28].

Lubrication Conditions	No Lubrication	Oil Lubrication	Thin-Film Lubrication
Friction coefficients	0.1~0.15~	0.1~0.12	0.08~0.12

## Data Availability

Not applicable.
